# Biosynthesis and Extracellular Concentrations of N,N-dimethyltryptamine (DMT) in Mammalian Brain

**DOI:** 10.1038/s41598-019-45812-w

**Published:** 2019-06-27

**Authors:** Jon G. Dean, Tiecheng Liu, Sean Huff, Ben Sheler, Steven A. Barker, Rick J. Strassman, Michael M. Wang, Jimo Borjigin

**Affiliations:** 1 Department of Molecular and Integrative Physiology, University of Michigan, Ann Arbor, MI USA; 2 Department of Comparative Biomedical Sciences, School of Veterinary Medicine, Louisiana State University, Baton Rouge, LA USA; 3 Department of Psychiatry, University of New Mexico School of Medicine, Albuquerque, NM USA; 4 Department of Neurology, University of Michigan, Ann Arbor, MI USA; 5 Neuroscience Graduate Program, University of Michigan, Ann Arbor, MI USA; 6 VA Ann Arbor Healthcare System, Ann Arbor, MI USA; 7 Center for Consciousness Science, University of Michigan, Ann Arbor, MI USA

**Keywords:** Neurochemistry, RNA, Transferases, Immunochemistry, Molecular neuroscience

## Abstract

*N,N*-dimethyltryptamine (DMT), a psychedelic compound identified endogenously in mammals, is biosynthesized by aromatic-*L*-amino acid decarboxylase (AADC) and indolethylamine-*N*-methyltransferase (INMT). Whether DMT is biosynthesized in the mammalian brain is unknown. We investigated brain expression of INMT transcript in rats and humans, co-expression of INMT and AADC mRNA in rat brain and periphery, and brain concentrations of DMT in rats. INMT transcripts were identified in the cerebral cortex, pineal gland, and choroid plexus of both rats and humans via *in situ* hybridization. Notably, INMT mRNA was colocalized with AADC transcript in rat brain tissues, in contrast to rat peripheral tissues where there existed little overlapping expression of INMT with AADC transcripts. Additionally, extracellular concentrations of DMT in the cerebral cortex of normal behaving rats, with or without the pineal gland, were similar to those of canonical monoamine neurotransmitters including serotonin. A significant increase of DMT levels in the rat visual cortex was observed following induction of experimental cardiac arrest, a finding independent of an intact pineal gland. These results show for the first time that the rat brain is capable of synthesizing and releasing DMT at concentrations comparable to known monoamine neurotransmitters and raise the possibility that this phenomenon may occur similarly in human brains.

## Introduction

*N,N*-dimethyltryptamine (DMT) belongs to a class of serotonergic psychedelics that includes lysergic acid diethylamide (LSD) and psilocybin^1^. DMT, like all serotonergic psychedelics, reliably elicits a wide spectrum of subjective effects on brain functions including perception, affect, and cognition^2^. These compounds share structural and functional similarities with serotonin (5-hydroxytryptamine, 5-HT), and interact with 5-HT and other receptors to produce their effects^3–5^. Unlike other psychedelics, however, DMT is endogenously produced in animals^6–8^, including humans^9–11^. In addition to the subjective psychedelic effects exogenous administration of DMT has on conscious experience, it has other well-documented anti-hypoxic^12^, antidepressant^13^, and plasticity-promoting actions^14^. Taking these facts together, a further understanding of why DMT is present in mammals is of interest.

Biosynthesis of DMT from tryptamine requires double methylation reactions catalyzed by indolethylamine-*N*-methyltransferase (INMT)^15,16^. INMT mRNA was identified at high levels in peripheral tissues in rabbits^17^ and in humans^18^. However, this peripheral INMT also methylates other ligands such as histamine^17,19^. In the brain, INMT mRNA was found at very low levels in rabbits^17^ and was undetectable in humans^18^. No study to date has yet identified INMT in the cerebral cortex in any species. In addition to INMT, production of DMT requires aromatic-*L*-amino acid decarboxylase (AADC), which removes the carboxyl group from dietary tryptophan to form tryptamine, the essential DMT precursor that can be rapidly metabolized by monoamine oxidase^20^. While high levels of INMT mRNA expression in the periphery^17,18^ have been assumed to indicate the potential for correspondingly high levels of DMT, cellular colocalization of INMT and AADC transcripts has not yet been reported in any tissue. Moreover, studies to date assessing levels of DMT in human peripheral bodily fluids have only reported it in trace amounts^11^, calling into question any physiological role.

In order to address some of these issues, this study examined expression of INMT mRNA in rat and human brain tissues using *in situ* hybridization. In addition, we conducted double *in situ* hybridization studies to probe the co-expression of INMT and AADC mRNA in rat brain and periphery. To investigate the presence of DMT in rat brain, we analyzed DMT content in microdialysis samples collected directly from the visual cortices of living pineal-intact and pinealectomized rats. Pinealectomized animals were studied because previous data established the presence of DMT in living rat pineal dialysate^8^. To investigate whether DMT brain levels are inducible by physiological alterations, we assessed its levels in rat brain following cardiac arrest with or without the pineal gland, as a prior study from our lab demonstrated a surge in the levels of select neurotransmitters in rat visual cortex following cardiac arrest using this technique^21^.

## Results

### INMT mRNA is expressed in rat and human brain

**Figure 1:**
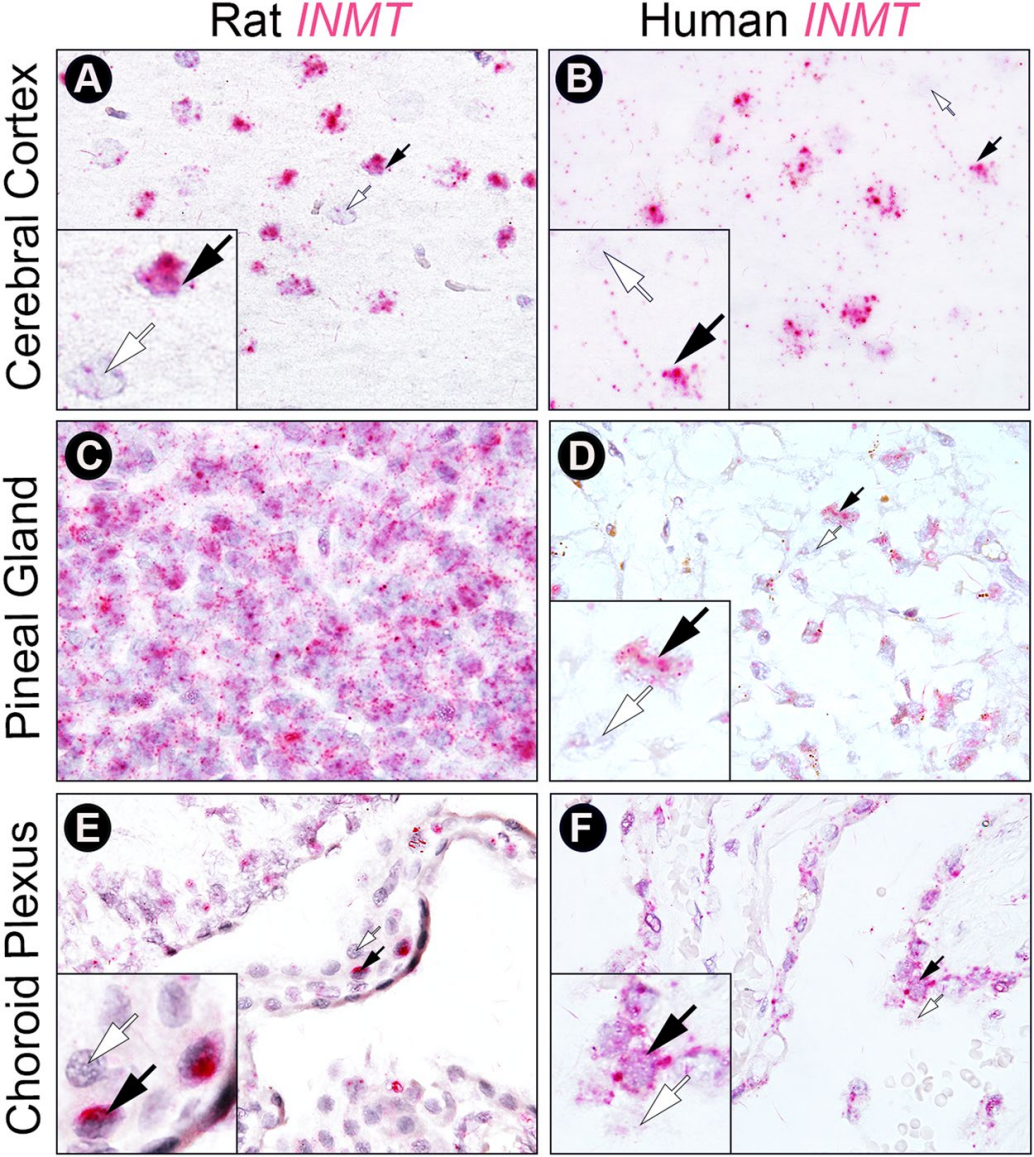
INMT mRNA is expressed in the brain. Images show results of *in situ* hybridization of INMT mRNA probes on rat (first column) or human (second column) brain FFPE sections. Where applicable, small black and white arrows denote exemplary cells positive and negative for INMT mRNA expression, respectively. Insets are magnifications of these cells. INMT mRNA expression is seen as pink punctate dots in cortical cells within rat visual cortex (**A**), in cells of the human medial frontal cerebral cortex (**B**), in pinealocytes within rat (**C**) and human (**D**) pineal gland, and in select ependymal cells of rat (**E**) and human (**F**) choroid plexus. In this and the additional *in situ* panels in Figs 2 and 3, nuclear counterstain (blue/gray staining in all images) identifies all cells/nuclei = 50% hematoxylin. All large panel images = 100x oil magnification. Histology sections are all coronal.

To investigate the cortical localization of INMT transcripts, we first conducted *in situ* hybridization for INMT mRNA on formalin-fixed paraffin-embedded (FFPE) rat brain tissue sections (Fig. 1). INMT mRNA, stained red/pink for the single staining *in situ* experiments, was consistently detected in rat visual cortex (panel A), human medial frontal cortex (panel B), rat (panel C) and human (panel D) pineal gland, and rat (panel E) and human (panel F) choroid plexus.

### INMT and AADC mRNA colocalize in rat brain tissues

**Figure 2:**
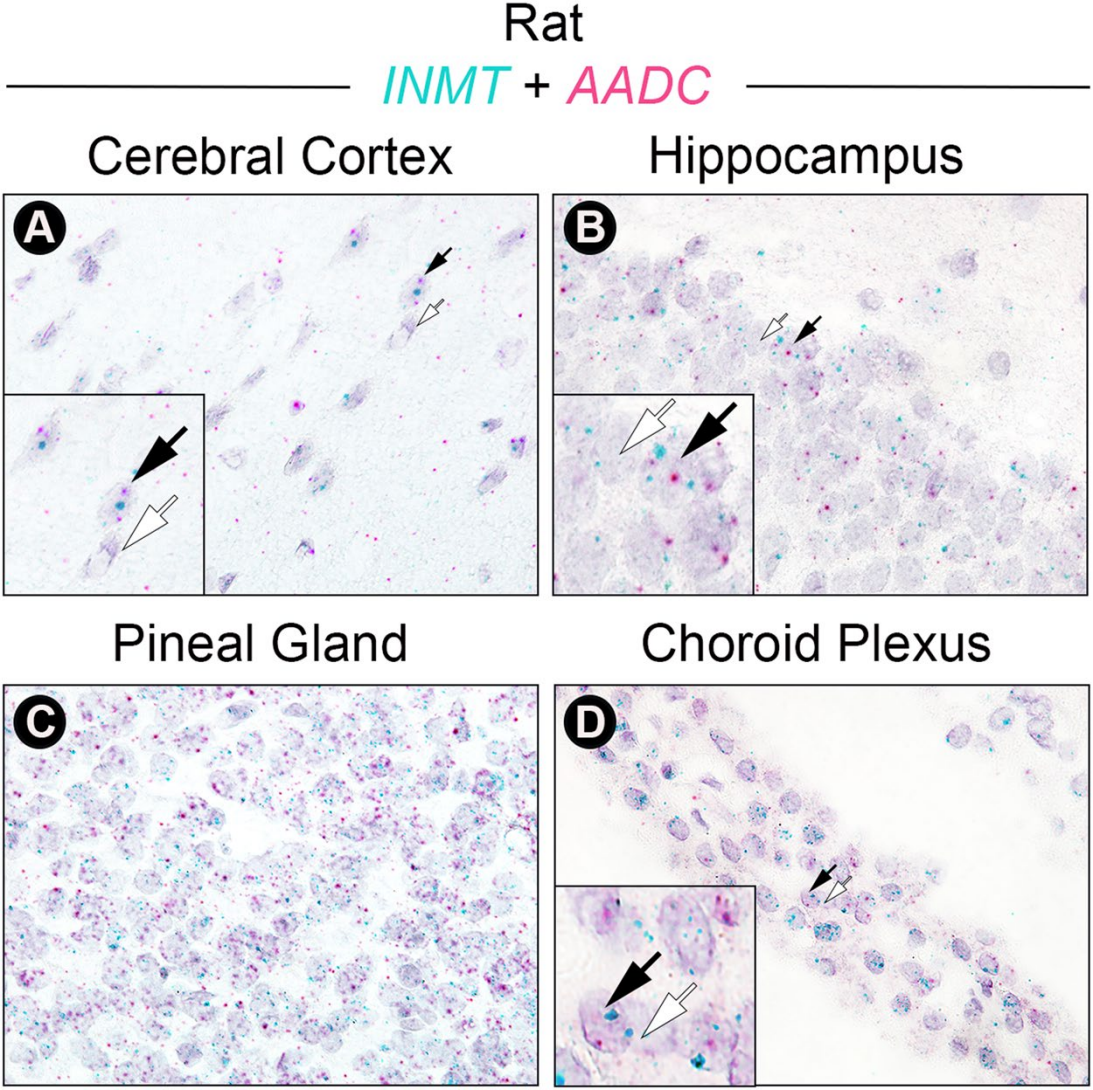
INMT mRNA is colocalized with AADC mRNA in the brain. Images show results of *in situ* hybridization on rat brain FFPE sections using INMT (green) and AADC (pink) mRNA probes. (**A**) INMT and AADC mRNA are colocalized in cells of the visual cortex (panel A), in cells of the hippocampus (**B**), in the pinealocytes (**C**), and in the ependymal cells of the choroid plexus (**D**).

A series of double *in situ* hybridization experiments was conducted on rat brain tissue sections (Fig. 2), adjacent to those used for single *in situ* hybridization (Fig. 1), to assess cellular colocalization of INMT with AADC transcripts. INMT (green) and AADC (red) mRNA colocalized extensively within the visual cortex (panel A), the hippocampus (panel B), the pineal gland (panel C), and the choroid plexus (panel D).

A series of control experiments using positive and negative control probes was conducted to assess the *in situ* technique, as well as to sample mRNA quality. As shown in Fig. S1, positive control probes (targeting *PPIB* and *POLR2A*, see Supplemental Information) produced the expected staining patterns in each of the rat brain sections (Fig. S1A,D,G,J), adjacent to those used in Fig. 2A–D; this demonstrates proper preparation of the rat brain FFPE sections. As expected, the negative control probe (a bacteria-specific gene, *dapB*) showed no appreciable staining (Fig. S1B,E,H,K) in the rat brain sections adjacent to those in Fig. 2A–D. Quantification of mRNA signal strength (calculated as fraction of total area in pixels) in Figs 2 and S1 images showed that the summated INMT and AADC mRNA signals in each rat brain tissue section were comparable to that of the positive control probes and well above negative control staining levels (Fig. S1C,F,I,L), thereby confirming the quality of the INMT and AADC mRNA signals following the chromogenic double *in situ* procedures. These data provide validation of the duplex *in situ* technique used to demonstrate the cellular colocalization of INMT and AADC mRNA (Fig. 2).

### INMT and AADC mRNA are sparsely colocalized in rat peripheral tissues

**Figure 3:**
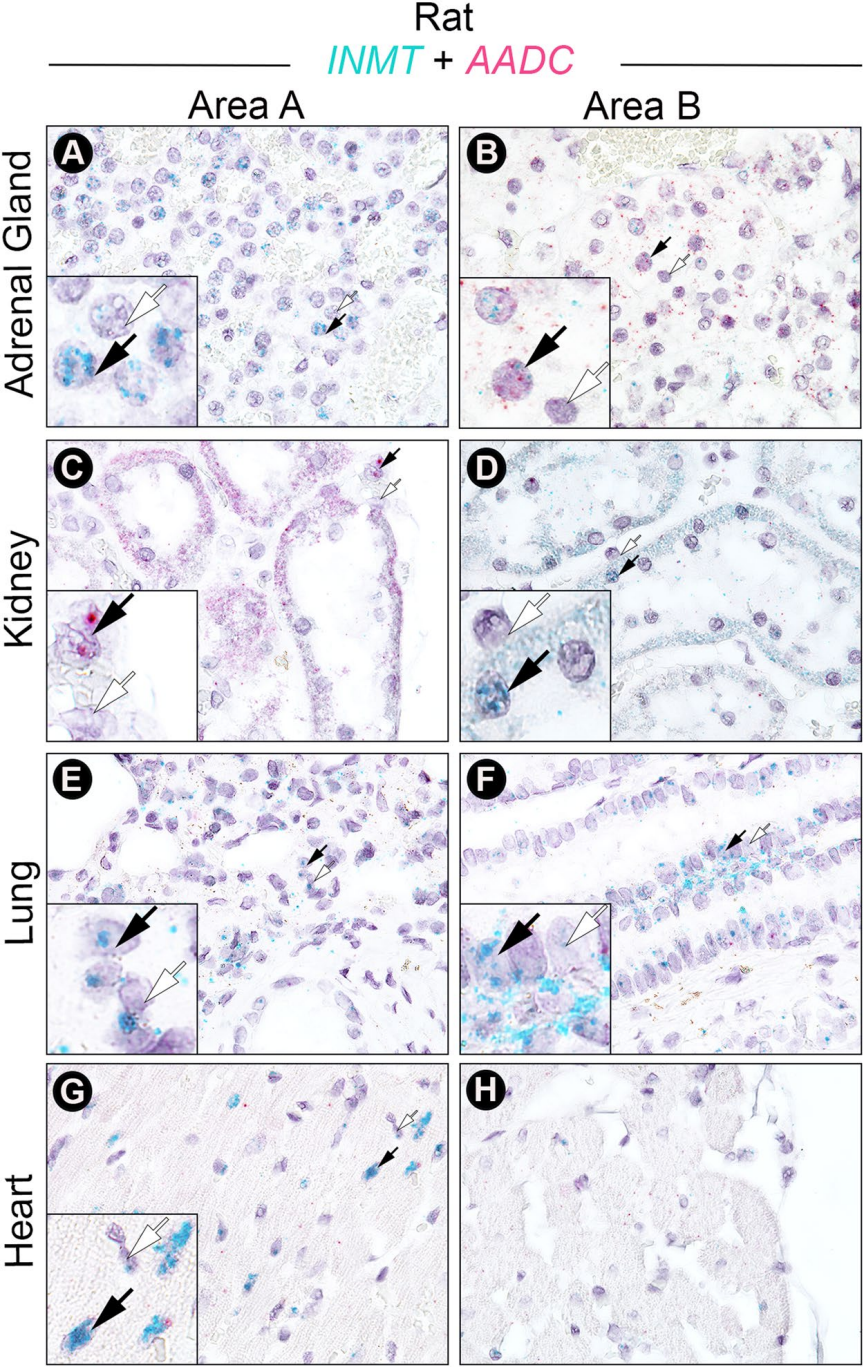
INMT and AADC mRNA expression is largely non-overlapping in rat peripheral tissues. Two representative regions (Area A and Area B) are shown for each tissue type. Insets show magnifications of areas of the main images wherein cells are positive (black arrows) or negative (white arrows) for either INMT (**A**,**D**–**G**), AADC (**C**), or both INMT and AADC (**B**) transcripts. INMT mRNA (green punctate dots) was highly expressed in the adrenal cortex (**A**), the renal tubules of the kidney medulla (**D**), the lung (**E**,**F**), and the heart (**G**). INMT transcripts were not uniformly distributed in these tissues, as INMT is found at very low levels in the adrenal medulla (**B**), renal cortex (**C**), and select areas of the heart (**H**). AADC mRNA (pink punctate dots) was expressed in adrenal medulla (**B**), where it is colocalized with INMT mRNA in select cells (black arrows in **B**), and in renal cortex (**C**) where it is not found together with INMT in the same cells. In the lung (**E**,**F**) and heart (**G**,**H**), AADC mRNA was nearly absent.

The *in situ* hybridization duplex assay was repeated on rat peripheral tissues including adrenal, kidney, lung, and heart (Fig. 3). While INMT mRNA was found at varying levels in all tissues tested (Fig. 3), AADC mRNA was only detectable in a subset of these tissues. The adrenal cortex was positive for INMT but not for AADC transcript (panel A), whereas the adrenal medulla stained more abundantly for AADC mRNA, with INMT transcript staining noted in only some cells (panel B). This pattern of adrenal medulla-specific AADC mRNA localization accords with previous literature^22,23^. Colocalization for INMT and AADC mRNA in rat adrenal gland was sparse, detected within select cells in adrenal medulla only (black arrows in panel B). INMT and AADC transcripts were both expressed in renal tubules in the kidney (panels C and D), but they were compartmentalized to discrete anatomical locales. Consistent with previous literature^22,23^, AADC mRNA expression was restricted to the renal cortex (panel C) with no appreciable expression observable in the kidney medulla (panel D). In clear contrast to AADC mRNA expression, INMT mRNA was localized to the kidney medulla (panel D) rather than the cortex (panel C). INMT mRNA was abundantly found in the lung, but AADC mRNA expression was scant (panels E and F), in accord with previous studies assessing AADC mRNA in rodent lung tissues^23^. Very few cells in rat lung tissues were found to express both INMT and AADC transcripts (panels E and F). Likewise, INMT mRNA was highly expressed in rat heart in selected areas (panel G), as noted in previous Northern blot data reported in humans^18^, but AADC expression was not observed in appreciable levels (panels G and H), consistent with a prior study in rodents^23^.

### DMT is present in the brain at concentrations comparable to known monoamine neurotransmitters

**Figure 4:**
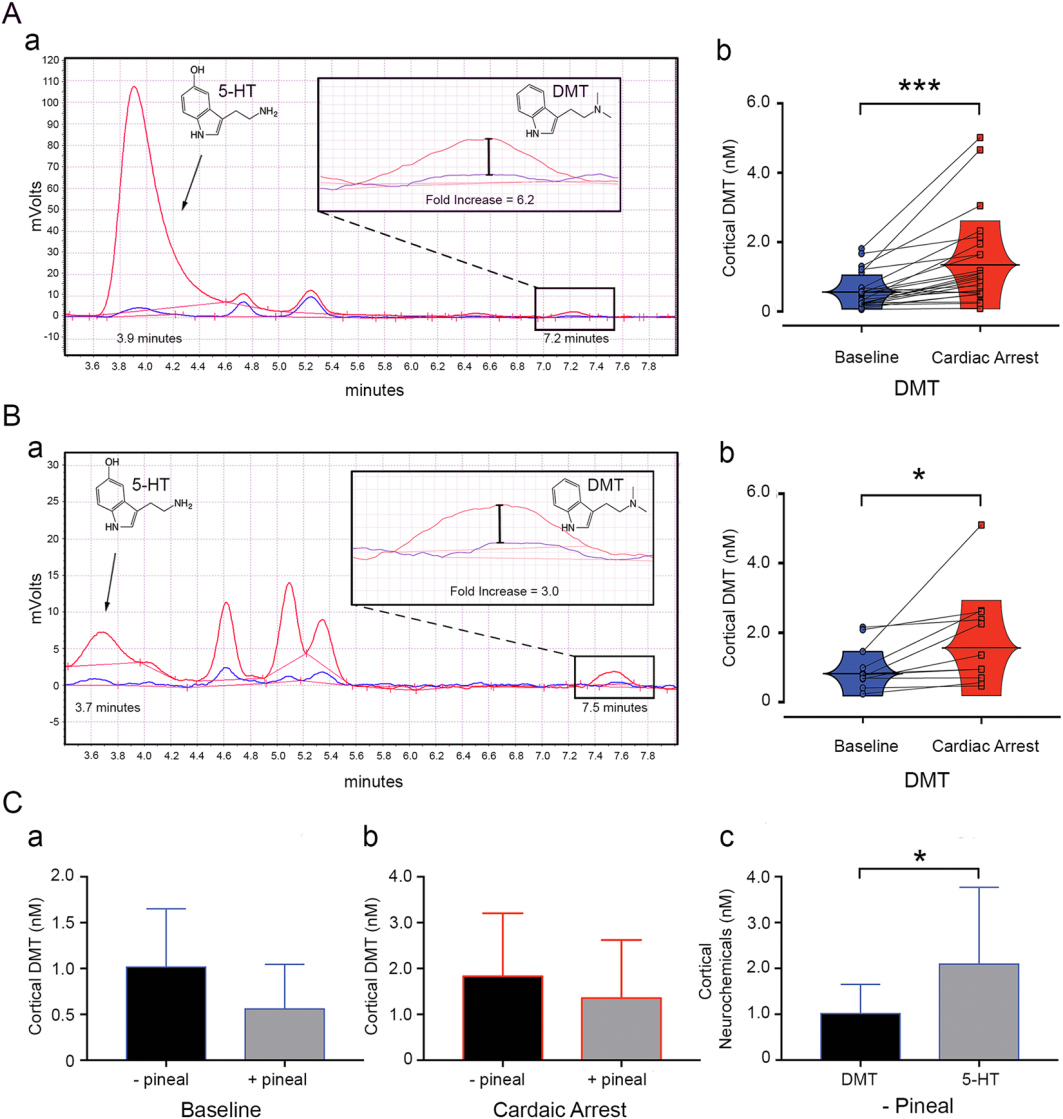
DMT is present in the brain at concentrations comparable to known monoamine neurotransmitters. (**A**) DMT levels in pineal-intact rats (n = 25) rose following cardiac arrest. DMT, sampled 0.5 hr before cardiac arrest shown (retention time = 7.2 minutes) on a representative raw chromatogram (a, blue tracing), ranged between 0.05–1.8 nM (averaged 0.56 nM; b, blue dots) during baseline. DMT levels increased within one hr of cardiac arrest (a, red tracing), averaging 1.35 nM with maximum 5.01 nM (b, red dots). DMT increase was significant (***p = 0.00027; mean of differences = 0.80; SD = 0.93; 95% CI = 0.41 to 1.18; t(24) = −4.26). (**B**) DMT levels persisted in brains of pinealectomized rats (n = 11). DMT levels sampled 0.5–1 hr following cardiac arrest shown (retention time = 7.5 minutes) on a representative raw chromatogram (a, blue tracing), ranged between 0.25–2.2 nM (averaged 1.02 nM; b, blue dots) during baseline. DMT levels increased within one hr of cardiac arrest (a, red tracing), averaging 1.83 nM with maximum of 5.11 nM (b, red dots). DMT increase was significant (*p = 0.034; mean of differences = 0.81; SD = 1.10; 95% CI = 0.075 to 1.55; t(10) = 2.45). (**C**) There was no difference in brain concentrations of DMT at baseline (a) between rats without (−pineal; mean = 1.02 nM; SD, blue bar = 0.63) and with ( + pineal; mean = 0.56 nM; SD, blue bar = 0.49) the pineal gland (p = 0.05, Welch’s unpaired t-test). No difference between brain concentrations of DMT following cardiac arrest (b) without (− pineal; mean = 1.83 nM; SD, red bar = 1.38) and with (+pineal; mean = 1.35 nM; SD, red bar = 1.27) the pineal gland was detected (p = 0.34, unpaired t-test with Welch’s correction). Baseline levels of DMT were about half those of 5-HT levels (c; *p = 0.026; mean of differences = 1.08; SD = 1.37; 95% CI = 0.16 to 2.0; t(10) = −2.62) in the visual cortex of pinealectomized rats (mean DMT = 1.02 nM; SD, blue bar = 0.63; mean 5-HT = 2.10 nM; SD, blue bar = 1.67).

To determine if DMT is present in the brain, we quantified rat brain microdialysates by conventional fluorescence detection coupled to a HPLC system (Fig. 4), a method that has been used in our laboratory to detect physiological neurochemicals like 5-HT and melatonin^24^. DMT as well as 5-HT were detected in rat brain (Fig. 4) in microdialysates collected from the visual cortex via this technique^24^. Under baseline conditions, the normal rat brain contained detectable levels of DMT (seen at retention time of 7.2 min; blue tracing in panel Aa), ranging 0.05–1.8 nM with an average of 0.56 nM (blue dots in panel Ab).

In our previous report of DMT in the rat brain^8^, it was unclear whether the detected DMT was from the cerebral cortex, pineal gland, or both, since the microdialysis probe traversed the rat brain through both the cortex as well as the pineal gland (see probe design in^24^). To determine the contribution of the cortex to DMT secretion, we compared normal rats (panel A) to pineolectomized animals (panel B). DMT in rats without the pineal gland, still detectable under baseline (seen at retention time of 7.5 min; blue tracing in panel Ba), ranged 0.25–2.2 nM with an average of 1.02 nM (blue dots in panel Bb). Cortical levels of DMT did not show significant difference between rats with and without the pineal gland under baseline conditions (panel Ca; p = 0.05, via unpaired t-tests with Welch’s correction).

To determine if DMT levels in the rat brain are regulated by physiological perturbations, we monitored the brain concentration of DMT before and after experimentally-induced cardiac arrest. Several hours of DMT baseline levels were first established in the brains of freely behaving and pineal-intact (panel A) and pinealectomized rats (panel B). DMT concentrations following cardiac arrest (red tracings) increased significantly over baseline (blue tracings) in both brains of the pineal-intact group (panel Ab; p = 0.00027) and the pinealectomized group (panel Bb; p = 0.034). We found that 60% (15/25) of normal pineal-intact rats demonstrated more than two-fold increases in DMT while 32% (8/25) showed less than 1.5-fold change (panel Ab). Of the rats without the pineal gland, 45% (5/11) showed more than two-fold increase in DMT whereas 55% (6/11) of rats exhibited minimum change (<1.5-fold; panel Bb) following the onset of cardiac arrest. Average fold increase following cardiac arrest for DMT in the brain was 2.84 ± 1.65 for the pineal-intact group (with a maximum of 6.18-fold), and 1.84 ± 0.89 for the pinealectomized group (with a maximum of 3.47-fold). DMT concentrations in rats exposed to experimentally-induced cardiac arrest did not differ significantly between pineal-intact and pinealectomized rats (panel Cb; p = 0.34 via unpaired t-tests with Welch’s correction).

To probe the relative abundance of extracellular DMT compared to 5-HT in cortical dialysates from the same rats, we quantified brain 5-HT, a monoamine neurotransmitter structurally related to DMT, in rats without the pineal gland. 5-HT was clearly detectable at baseline in the rat cerebral cortex in the absence of the pineal gland (panel Ba). Mean extracellular concentration of 5-HT was 2.10 ± 1.67 nM in these rat brain dialysates (panel Cc). In comparison, basal DMT levels were 1.02 ± 0.63 nM from the same cortical dialysates (panel Cc). Although DMT concentrations are lower than those of 5-HT levels (Fig. 4Cc) under basal conditions, they are comparable to the extracellular levels of all known monoamine neurotransmitters measured by *in vivo* microdialysis in rat brain^25^.

## Discussion

We report [1] cortical expression of INMT mRNA in rat and human brain, [2] colocalization of INMT and AADC mRNA in the same cells in rat brain, and [3] predominately non-overlapping expression of INMT with AADC mRNA in rat peripheral organs including the adrenal, kidney, lung, and heart. We further show that DMT is present in rat visual cortex in pineal-intact and pinealectomized animals. Moreover, we show DMT levels are significantly elevated by experimentally-induced cardiac arrest. Collectively, these data support the notion that DMT is synthesized in rat brain and at concentrations consistent with that of other known monoamine neurotransmitters. Our demonstration of INMT mRNA expression in human cerebral cortex, choroid plexus, and pineal gland also suggest that DMT biosynthesis may similarly occur in the human brain.

This study demonstrates for the first time that INMT mRNA, the transcript for a key DMT synthetic enzyme, is widely expressed in the cerebral cortex in rats. Importantly, INMT and AADC transcripts are co-expressed in the same brain cells, providing a plausible mechanism for cellular synthesis of DMT in the mammalian neocortex. DMT is a monoamine produced from tryptophan. If endogenous DMT functions as a non-classical monoamine neurotransmitter in the brain, DMT would be the only monoamine whose biosynthesis takes place within the cerebral cortex where it may directly influence cognitive functions of the brain.

While AADC expression in the brain is well known^26^, INMT expression in the brain was unclear prior to our study. Earlier studies showed that INMT mRNA was only weakly present in rabbit brain tissues^17^, and undetectable in human brain tissues^18^. These prior reports are all based on Northern blot analysis, which provides no information on cellular distribution of INMT mRNA and is less sensitive compared to quantitative polymerase chain reaction or the *in situ* hybridization method used in our study. We were able to identify INMT mRNA in both rat and human brain tissues using the RNAscope *in situ* assay system, in part because of the high sensitivity afforded by this technique^27^, which allowed robust and unambiguous identification of INMT mRNA in both rat and human brain FFPE tissues for the first time (see Fig. 1).

AADC is best characterized as one of the enzymes necessary for the synthesis of all canonical monoamine neurotransmitters, including 5-HT [together with tryptophan hydroxylase (TPH)] and dopamine/norepinephrine [together with tyrosine hydroxylase (TH)]. In the pineal gland, AADC (together with TPH) is well known to mediate the synthesis of 5-HT, the precursor for melatonin production^28^. In the brain, AADC mRNA is present abundantly in the monoamine neurons in the brainstem^26^. However, AADC mRNA and protein have also been reported in neurons of the cerebral cortex and hippocampus^26^ that do not contain TH/TPH in mice, rats and humans^29–33^. To date, the function of these AADC-positive and TH/TPH-negative neurons is unknown.

Using the RNAscope *in situ* hybridization technique, we identified AADC mRNA in several regions of the rat brain (Fig. 2), including the cerebral cortex and hippocampus (Fig. 2), consistent with what has been reported in mouse brain^26^. Critically, we found in each of these rat structures that AADC mRNA is colocalized with an INMT transcript (Fig. 2), suggesting that these INMT-positive and AADC-positive neurons may function as DMT-producing neurons (or D-neurons) and that DMT, a non-canonical monoamine, may possibly be the principle neurotransmitter in these widely distributed brain D-neurons. A functional role of the D-neurons will need to be explored in future studies. For example, INMT-deficient animals are necessary to demonstrate unequivocally that INMT is responsible for the endogenous production of DMT.

INMT protein was also found in monkey pineal gland in a prior study^34^. Our study expanded this finding and demonstrated an abundant expression of INMT mRNA in the pineal gland in both rats and humans (Fig. 1). Co-expression of INMT and AADC mRNA in rat pinealocytes was confirmed by double *in situ* hybridization analysis (Fig. 2), suggesting that a potential mechanism for DMT biosynthesis, as in the rat visual cortex, exists in the pineal gland. Transcripts for DMT synthetic enzymes were detected additionally in the choroid plexus, a venous network lining the ventricles of the brain that produce CSF, a fluid in which DMT has been detected^11^. The choroid plexus contains a high density of 5-HT2C receptors whose stimulation by agonists leads to marked reduction of CSF production^35^. As DMT is an agonist at the 5-HT2C receptor^4,5,36^ and LSD binds to the 5-HT2C receptor in cultured choroid plexus epithelial cells to stimulate their activity^37^, the effect of endogenous DMT on CSF regulation deserves additional investigation.

The high levels of INMT mRNA previously found in peripheral tissues^18^ have led to speculation that DMT is released from peripheral stores and subsequently transported to the brain. Consistent with earlier Northern blot analysis of human tissues^18^, we found high levels of INMT mRNA expression in rat kidney, lung, heart, and adrenal (Fig. 3). However, INMT mRNA expression in rat peripheral tissues showed very limited overlap with AADC mRNA at the cellular level, suggesting that INMT may have functions independent of DMT synthesis in the periphery. Although our present data suggest that DMT in the brain does not originate from peripheral sources but, rather, is produced locally in specific brain tissues, future studies (using brain-specific INMT knockout animals, for example) could be conducted to conclusively demonstrate that DMT found in brain dialysates originates from the brain. Peripheral INMT may be responsible for established DMT-independent functions such as methylation of sulfur-containing compounds^38^, histamine^17,19^, and selenium metabolism^39^.

DMT is an endogenous monoamine whose physiological functions remain unknown. Furthermore, since exogenous DMT can bind with nanomolar affinities to various receptors including 5-HT receptors^40–43^ and trace-amine associated receptors (reviewed in ref.^44^), endogenous DMT may influence brain functions via the same receptors. If DMT were indeed to function as a non-canonical monoamine neurotransmitter, however, it must be present in the brain at a physiologically relevant concentration. Presence of DMT has been reported in various species including rats^6–11^, yet its *in vivo* concentration in living brain has not been reported. This study represents the first quantification of DMT in the extracellular fluid of the brain in freely moving and normal behaving animals.

The baseline concentration of DMT in cortical microdialysates ranged between 0.05 to 1.8 nM (blue dots in Fig. 4Ab) in pineal-intact rats, and between 0.25 to 2.2 nM (blue dots in Fig. 4Bb) in pinealectomized rats. DMT levels showed no significant difference between rats with the pineal and without the pineal gland (Fig. 4Ca). Importantly, this cortical concentration of DMT (average 1.02 nM) is only slightly lower than that of 5-HT detected in the same dialysates, which averaged around 2 nM in rats without the pineal gland (Fig. 4Cc). This value of 5-HT in brain dialysates is consistent with what others have found in rats (average 0.87 nM; 0.12–3.4 nM range) [reviewed in ref.^25^]. In fact, the basal DMT concentrations (Fig. 4Ab; average 1.02 nM; 0.25–2.2 nM range) in the brain microdialysates are well within the known range of all three canonical monoamine neurotransmitters^25^: 5-HT (average 0.87 nM; 0.12–3.4 nM range), norepinephrine (average 1.77 nM; 0.19–4.4 nM range), and dopamine (average 1.5 nM; 0.07–4.9 nM range). We wish to emphasize that our microdialysates were collected and analyzed online in real time from rats without any pretreatment to block the activity of monoamine oxidase, an enzyme that rapidly degrades DMT *in vivo*^15,45^. The mechanism and function of cortical DMT production remain to be fully investigated.

DMT was previously detected in freely moving rats^8^. However, DMT was not quantified in that study. Furthermore, the microdialysates used for DMT analysis^8^ were collected from both the pineal gland and the surrounding visual cortex of rats and it was unclear whether the detected DMT was from the pineal alone, the cortex alone, or both. To address this latter issue, we surgically removed the pineal gland and monitored DMT levels in rats both under baseline and following cardiac arrest (Fig. 4B). We found that DMT concentrations in the brain are independent of the pineal gland, as cortical DMT levels show no significant difference with or without the pineal gland (Fig. 4Ca). These data suggest that the cortex may be one of the major sources of released DMT from the brain. At this point, it is uncertain to what extent, if any, the pineal contributes to DMT production^8^. It is also unclear whether DMT detected in the brain extracellular fluid is secreted from neurons and/or glial cells. Colocalization studies using both an INMT probe and cell-type specific markers should be used in future studies to clarify this.

The extensive colocalization of INMT and AADC transcripts in pinealocytes (Fig. 2C), and the apparent lack of the pineal’s contribution to the brain production of DMT (Fig. 4Ca) present a paradox. We reasoned that there may be two competing pathways for tryptophan in the pineal: (1) conversion of tryptophan to 5-HT via sequential actions of TPH and AADC, and (2) the potential conversion of tryptophan to DMT via sequential actions of AADC and INMT. It is plausible that in the pineal, the first pathway is overwhelmingly dominant, resulting in high production of 5-HT at the expense of DMT. The established properties of the enzymes support this: the affinity of tryptophan for TPH (*K*_m_ = 41.3 uM)^46^ in the 5-HT/melatonin synthesis pathway is substantially higher compared to the reported affinity of tryptophan for AADC (*K*_m_ = 3000 uM)^47^ in the DMT biosynthetic pathway. Thus, if the same cell expresses both TPH and AADC, tryptophan will be more likely to be converted to 5-hydroxytryptophyan (destined to 5-HT) than to tryptamine (destined to DMT). Additionally, 5-hydroxytryptophan binds to AADC with substantially higher affinity than tryptophan (*K*_m_ = 160 uM^48^ versus *K*_m_ = 3000 uM)^47^. As a result, high levels of 5-hydroxytryptophan found in the pineal are likely to competitively inhibit AADC conversion of tryptophan to tryptamine, further reducing this intermediate in DMT production. The relatively low affinity of AADC for tryptophan thus reduces the production of DMT in pineal cells that contain both TPH and AADC. These data may explain the possibility that the pineal gland contributes little to DMT production, despite the abundant co-expression of AADC and INMT in the pinealocytes. We could not, however, rule out the possibility that there may be compensatory increases in DMT’s biosynthesis in other brain areas following the loss of the pineal gland.

In our previous studies, we have observed a marked elevation of some, but not all, critical neurotransmitters in rat brain during asphyxic cardiac arrest^21^, which we posit may contribute to the elevated conscious information processing observed in dying rats^21,49^. These data also suggest that global ischemia (by cardiac arrest, as in the current study), similar to global hypoxia (by asphyxia, as in^21^), leads to a tightly regulated release of a select set of neurotransmitters^21^. To test whether DMT concentrations are regulated by physiological alterations, we monitored DMT levels in rat brain dialysates following experimentally-induced cardiac arrest, and identified a significant rise in DMT levels in animals with (Fig. 4A) and without the pineal (Fig. 4B).

The cardiac arrest-induced increase of endogenous DMT release may be related to near-death experiences (NDEs), as a recent study reports NDE-like mental states in human subjects given exogenous DMT^50^. Not all rats in our current study exhibited a surge of DMT following cardiac arrest (Fig. 4), an interesting observation in light of the fact that NDEs are reported by less than 20% of patients who survive cardiac arrests^51^. It is unknown whether the concentrations of DMT reported in our study at cardiac arrest can elicit the effects of an exogenous psychedelic dose of DMT, or whether this surge of endogenous DMT similarly occurs in humans. Moreover, the conscious states reported by NDE survivors may involve contributions from several of the other neurotransmitters found to surge at cardiac arrest in our prior rodent study^21^. Further investigation is clearly warranted to investigate whether DMT plays a role in generating neural correlates of near-death consciousness.

In conclusion, we present evidence for the brain expression of mRNA for INMT, the key DMT synthetic enzyme previously thought to exist only in the periphery, and demonstrate that, unlike in the periphery, transcripts for the DMT synthetic enzymes AADC and INMT are co-expressed in the same cells within the rat brain. The wide co-expression of transcripts for the DMT synthetic enzymes in the rat brain and basal concentrations of DMT comparable to that of other monoamine neurotransmitters indicate that endogenous DMT may influence brain function.

## Materials and Methods

### Animals

Adult (2–5 months of age) male Wistar rats (Harlan Laboratories, Indianapolis, IN) were divided into two groups for *in vivo* microdialysis: rats with intact pineal glands (n = 25; average weight = 334 g) and pinealectomized rats (n = 11; average weight = 335 g). All animals were housed in an 12:12 light:dark cycle in a controlled light environment (250 Lux at the cage level). The 12 hours of light began at 6 am and the 12 hours of dark began at 6 pm. The animals were given food and water *ad libitum*. The study was approved by the Animal Care and Use Committee at the University of Michigan, Ann Arbor, Michigan. All methods were performed in accordance with the relevant guidelines and regulations laid down by the Committee.

### Rat formalin-fixed paraffin-embedded (FFPE) tissues

Following euthanasia, rat brains and peripheral organs were harvested immediately and perfused in 1:10 formalin for one week. Tissues were then placed into 70% ethanol, dissected, and sectioned to a thickness of 5 µm by the University of Michigan Cancer Histology Core.

### Human FFPE tissues

FFPE human tissues used in Fig. 1 were archived from the University of Michigan. All samples were obtained from human autopsies and de-identified and, as such, were deemed exempt for regulation by the Institutional Review Boards at the University of Michigan.

### INMT and AADC mRNA *in situ* hybridization

For single staining experiments, *in situ* probes for rat INMT (Catalog No. 418021) and human INMT (Catalog No. 459961) and the chromogenic RNAscope® 2.0 HD Detection Kit (RED) for FFPE tissues (an alkaline phosphatase system, Catalog No. 310036) were purchased from Advanced Cell Diagnostics (Hayward, CA, USA). Protocol was followed according to the RNAscope® Sample Preparation Pretreatment Guide for FFPE (Catalog No. 320511) and RNAscope® 2.0 HD Detection Kit (RED) User Manual Part 2 (Catalog No. 320487). INMT mRNA was stained red and counterstained with 50% hematoxylin. For double staining experiments, *in situ* positive control probes [ready to use (RTU) mixture of two probes targeting *PPIB* and *POLR2A* (Catalog No. 313921) and negative control probes (Catalog No. 310043 – *dapB*; RTU probe targeting a bacterial gene), as well as probes for rat INMT (Catalog No. 418021) and AADC (Catalog No. 428331-C2), and the RNAscope® 2.5 HD Duplex Detection Kit (Chromogenic) for FFPE (a mixed alkaline phosphatase and horseradish peroxidase system, Catalog No. 322430) were likewise purchased from Advanced Cell Diagnostics. Protocol was followed according to the FFPE Sample Preparation and Pretreatment User Manual for the RNAscope® 2.5 Assay Part 1 (Document No. 322452-USM) as well as the RNAscope® 2.5 HD Duplex Detection Kit (Chromogenic) User Manual Part 2 (Document No. 322500-USM). The only change in the protocol was to increase AMP 5 and AMP 9 incubation from 30 minutes to 1 hour per recommendation of Advanced Cell Diagnostics. INMT mRNA was stained green (horseradish peroxidase) while AADC mRNA was stained red (alkaline phosphatase). Single and double staining experiments were both completed in single day-long sessions. Slides were counterstained with 50% hematoxylin. Positive and negative control probes were run on samples in dual channels to assay for mRNA quality and background signal in samples, as well as to verify kit performance. Positive staining was defined by signal intensity stronger than that of the negative control. In general, small-punctate dots are taken to represent a single transcript whereas larger dark clusters represent multiple transcripts in close proximity, i.e., signal intensity is a qualitative measure of transcript level^27^. Semi-quantitative analysis of mRNA signal was conducted for duplex staining on rat brain experimental tissues for comparison to control images in all duplex images using ImageJ (Version 1.50i) as defined in the proceeding section. All images were captured on an Olympus BX-51 with a DP70 camera. Magnifications are specified in figure legends. Images were processed with Adobe Photoshop CC 2015.5 (Adobe Systems Inc.).

### Semi-quantitative analysis of mRNA duplex staining in rat brain tissues using ImageJ

Experimental rat brain tissue sections subjected to duplex staining for INMT and AADC mRNA and positive and negative control probes on adjacent brain sections (images in Figs 2 and S1) were quantified using ImageJ to estimate the fraction of total area in pixels of mRNA signals by summating both colormetric channel pixels as per a prior publication analyzing this variable in brightfield images of rat FFPE brain tissues following RNAscope *in situ*^52^. Results of mRNA quantification are presented in Fig. S1 (summated INMT and AADC signals for comparison of experimental versus control tissue sections). Briefly, blue channel staining was quantified in all Figs 2 and S1 images with the following Color Threshold command adjustments (under the Adjust command). All default settings were used, except hue was set for 0–163, saturation was set for 0 to 90–255, depending on background, and brightness was set for 0–215 to 255, depending on background. Similarly for red channel staining, all default settings were used, except HSB color space was changed to YUV, and Y was set for 0–135 to 200, depending on background, U was set for 0–255, and V was set for 135–185 to 255, depending on background. Particles were then analyzed using the Analyze Particles command and default settings therein along with the Summarize box option checked. Outputs from the Summarize box for %Area for both colormetric channels were entered into GraphPad Prism Version 7.0a for Mac OS X to construct graphs in Fig. S1. All images were processed beforehand with the same microscope settings and Adobe Photoshop CC 2015.5 (Adobe Systems Inc.) filters.

### Surgical implantation of microdialysis probes

Microdialysis probes were constructed as previously described^24^ and their surgical implantation was conducted on each rat (n = 36) for pineal and/or cortical dialysate collection using a method modified from a published protocol and under strictly aseptic conditions^24^. The rats were anesthetized lightly first using a combination of ketamine (10 mg//kg, i.m.) and xylazine (2 mg/kg, i.m.). The animal’s head was shaved and positioned in a stereotaxic instrument with the head flat. For the rest of the surgery, anesthesia was provided by 1.8% (1.5–2%) isoflurane. The skull was exposed by a 2 cm coronal incision between the two ears along the interaural line. Three stabilizing stainless steel screws 1 mm in diameter were placed to allow the positioning of the probes on the skull. Two small burr holes were created on both sides of the skull. The smaller hole on the right side was ~0.5 mm in diameter, which prevented the tip of the 25-gauge dialysis needle from penetrating the skull, whereas the larger hole on the left side was ~1 mm in diameter and allowed the probe to easily exit the skull during implantation. Next, the right probe was carefully pushed into the brain tissue through the pineal from the right side of the skull leaving the epoxy ball outside of the skull. Following the completion of probe insertion, the epoxy on the left side was removed using cautery and the tungsten rod was then carefully pulled out of the probe. The excess dialysis fiber was cut and the hollow fiber tip was then secured to the tip of the second part of the probe using epoxy resin. The probe setup was fixed to the anchor screws on top of the skull with dental cement. Finally, the skin was sutured, leaving two probes: one to introduce the perfusate, and the other end to collect dialysate. The entire procedure took less than two hours per animal. The animals were returned to their cages, housed individually, and allowed to recover from the surgery for 7 days before microdialysis recording proceeded. Target was verified via HPLC assessment of dialysate for melatonin, which emanates specifically from the pineal in the brain.

### Pinealectomy

The pineal gland was first surgically removed from 11 rats. After the pineal gland was removed from the brain, a microdialysis probe was then inserted into the pinealectomized visual cortex using the same coordinates we used when the pineal gland was intact^8^. Animals were allowed 7 days to recover before baseline microdialysis sampling began. Pineal removal was further confirmed, via HPLC, by the absence of melatonin in the dialysate.

### *In vivo* measurement of DMT secretion

An automated system combining microdialysis with real time HPLC analysis was utilized for measurement of DMT secretion in both normal (n = 25) and pinealectomized animals (n = 11) at baseline and following induction of cardiac arrest. The system is outlined with illustrations elsewhere^24^. DMT retention times were determined using reference standards, as in our previous publication^8^. The HPLC system consisted of one Shimadzu SCL-10A VP controller, two Shimadzu LC-20AD isocratic pumps, a CTO-20AC column oven containing 2 Supelco C18 reversed phase columns, two RF-10AXL detectors, two VICI Cheminert^®^ sample injectors (2-position/10-port actuator), and a VICI digital sequence programmer.

Each HPLC system was designed to analyze data simultaneously from 4 rats, with 2 rats using each detector. Following one week of recovery, animals were placed within a light-controlled microdialysis chamber that held 2 cages. Rats were housed at 250 lux on an light:dark cycle with a dark period of 18:00–6:00 and were allowed to move freely throughout due to a swivel mounted on a counterbalance arm. The 21-gauge needle base was connected with PEEK tubing and a syringe to link two rats each to an Instech peristaltic pump. Microdialysis was performed at a continuous 2 μL/min flow rate with an artificial cerebrospinal fluid (CSF) solution consisting of NaCl (148 mM), KCl (3 mM), CaCl_2_·2H_2_O (1.4 mM), MgCl_2_·6H_2_O (0.8 mM), Na_2_HPO_4_·7H_2_O (0.8 mM), and NaH_2_PO_4_·H_2_O (0.2 mM). Pineal/cortical dialysates were collected from two rats in 15 or 30 minute intervals and delivered to a sample loop (Instech, Plymouth Meeting, PA, USA) during which time previously collected samples for two rats were injected by a VICI Cheminert^®^ sample injector (2-position/10-port valve) into a reversed phase C18 column, 250 × 4.6 mmm with 5 μm packing (Sigma, St Louis, MO, USA) maintained at 45 °C. A Shimadzu LC-20AD isocratic pump (Shimadzu, Tokyo, Japan) delivered the mobile phase, which consisted of 34% methanol with 10 mM sodium acetate, at 1.5 mL per minute. Since DMT is naturally fluorescent, as are all other compounds derived from tryptophan, samples were analyzed online by a Shimadzu fluorescence detector (excitation: 280 nm; emission: 345 nm) without any derivatization. The automated control was carried out with an external computer using Shimadzu chromatography software.

Dialysates were collected in 15 or 30 minute intervals for several hours or days to establish baseline levels, at which point rats were euthanized by injection of 10% KCl to the heart between approximately 11:00–12:00. Content of DMT, melatonin, and 5-HT in the dialysates were identified using internal controls and quantified as previously described^8,24^. Data collection and sequence processing were performed on CLASS-VP firmware from Shimadzu. Baseline values for DMT were calculated as the average of 3 stable time points 2 hours prior to induction of cardiac arrest and compared to the maximum peak DMT value following induction of cardiac arrest. The automated analysis of these compounds at baseline and maximum points following induction of cardiac arrest was confirmed manually if necessary.

### Experimental design and statistical analysis

Statistical analysis was conducted using RStudio (Macintosh Version 0.99.903) for paired two-tailed t-tests and unpaired t-tests with Welch’s correction. Exact p-values and means, means of differences, standard deviations, 95% confidence intervals, t values, and degrees of freedom are reported throughout the manuscript and completely in Figure legends. All plots and graphs were created with GraphPad Prism Version 7.0a for Mac OS X (La Jolla, CA) and further edited in Adobe Photoshop CC 2015.5 and Illustrator CC 2015.3 (Adobe Systems Inc.).

## Supplementary information

Biosynthesis and Extracellular Concentrations of N,N-dimethyltryptamine (DMT) in Mammalian Brain

## Data Availability

Original data that support the findings of this study are available from the corresponding author (J.B.).
