# Supplementary material for: Biosynthesis and Extracellular Concentrations of N,N-dimethyltryptamine (DMT) in Mammalian Brain
Source: Sci Rep. 2019 Jun 27;9:9333. doi: 10.1038/s41598-019-45812-w (PMC6597727; doi:10.1038/s41598-019-45812-w)
Supplement: Supplementary file 1 — Biosynthesis and Extracellular Concentrations of N,N-dimethyltryptamine (DMT) in Mammalian Brain [file 41598_2019_45812_MOESM1_ESM.pdf]

Biosynthesis and Extracellular Concentrations of N,N-dimethyltryptamine (DMT) in Mammalian Brain

Jon Dean[1], Tiecheng Liu[1], Sean Huff[1], Ben Sheler[1], Steven A. Barker[2], Rick J. Strassman[3], Michael M. Wang[1,4,5,6], and Jimo Borjigin[1,4,5*]

Departments of [1]Molecular and Integrative Physiology and [4]Neurology, [5]Neuroscience Graduate Program, University of Michigan, Ann Arbor, MI, USA; [6]VA Ann Arbor Healthcare System, Ann Arbor, MI, USA; [2]Department of Comparative Biomedical Sciences, School of Veterinary Medicine, Louisiana State University, Baton Rouge, LA, USA; [3]Department of Psychiatry, University of New Mexico School of Medicine, Albuquerque, NM, USA

***Corresponding author:**
J Borjigin, PhD,
Department of Molecular and Integrative Physiology,
University of Michigan Medical School,
1137 East Catherine Street, 7732C MS II
Ann Arbor, MI 48109-5622, USA.
E-mail: Borjigin@umich.edu

Supporting Information

SI Figure Legends

Figure S1. Positive and negative controls versus experimental duplex staining. All positive controls are an RTU mixture of two probes targeting PPIB and POLR2A "housekeeping" genes and negative controls targeting *dapB*, a bacteria specific gene. *PPIB* is in the pink/red channel and *POLR2A* is in the green channel in all positive control images. **(A)** Positive control on rat visual cortex tissue section demonstrating sensitivity of the *in situ* procedure. Several brain cells can be seen double-positive (green and pink staining) for both probes. **(B)** Negative control on an adjacent section of rat visual cortex demonstrating absence of staining when the bacteria specific *in situ* probe is applied to the tissue. **(C)** Results from ImageJ analysis of summated fraction of total area (pixels^2) for both green INMT mRNA and pink AADC mRNA probe staining for the original rat visual cortex tissue image in Fig. 2A versus the staining for positive and negative control probes in (**A**) and (**B**) demonstrating similar positive staining signal strength and minimal off-target nonspecific staining (negative control). Similar results were found when these same positive and negative control probes were applied to and quantified versus experimental images in Figs. 2B-D for rat **(D-F)** hippocampal, **(G-I)** pineal and **(J-L)** choroid plexus tissues. Nuclear counterstain (blue/gray staining in all images) identifies all cells/nuclei = 50% hematoxylin. All images = 100x oil magnification. Histology sections are all coronal.

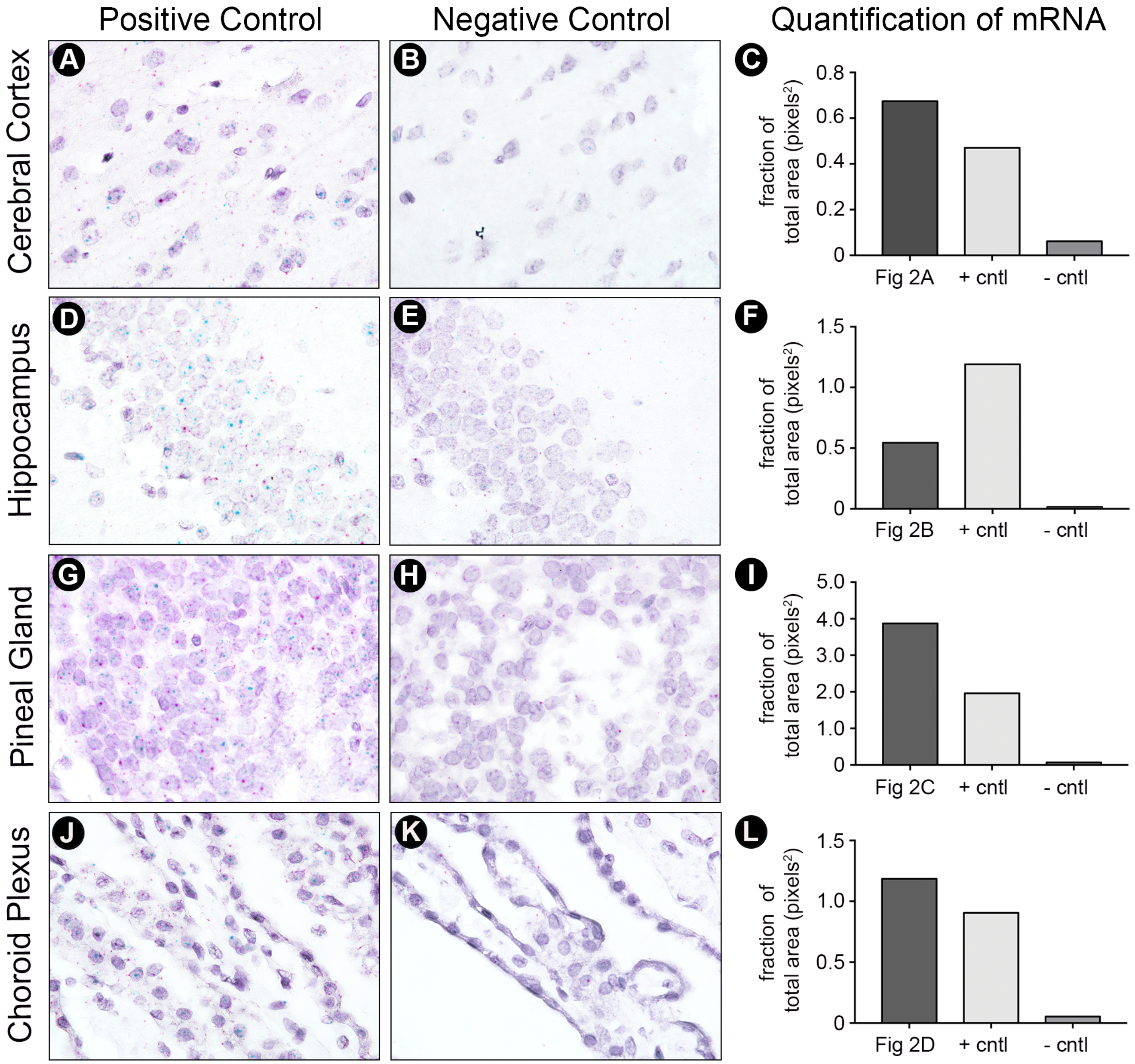
Positive Control
Negative Control
Quantification of mRNA
Cerebral Cortex
Hippocampus
Pineal Gland
Choroid Plexus
A
B
C
D
E
F
G
H
I
J
K
L
fraction of total area (pixels²)
Fig 2A
Fig 2B
Fig 2C
Fig 2D
+ cntl
- cntl
0
0.2
0.4
0.6
0.8
0.5
1.0
1.5
2.0
3.0
4.0
5.0
